# A plant virus protein, NIa-pro, interacts with Indole-3-acetic acid-amido synthetase, whose levels positively correlate with disease severity

**DOI:** 10.3389/fpls.2023.1112821

**Published:** 2023-09-11

**Authors:** Prabu Gnanasekaran, Ying Zhai, Hira Kamal, Andrei Smertenko, Hanu R. Pappu

**Affiliations:** ^1^ Department of Plant Pathology, Washington State University, Pullman, WA, United States; ^2^ Institute of Biological Chemistry, Washington State University, Pullman, WA, United States

**Keywords:** potato virus Y, auxin conjugation, indole-3-acetic acid-amido synthetase, auxin homeostasis, plant innate immunity

## Abstract

Potato virus Y (PVY) is an economically important plant pathogen that reduces the productivity of several host plants. To develop PVY-resistant cultivars, it is essential to identify the plant-PVY interactome and decipher the biological significance of those molecular interactions. We performed a yeast two-hybrid (Y2H) screen of *Nicotiana benthamiana* cDNA library using PVY-encoded NIa-pro as the bait. The *N. benthamiana Indole-3-acetic acid-amido synthetase* (IAAS) was identified as an interactor of NIa-pro protein. The interaction was confirmed via targeted Y2H and bimolecular fluorescence complementation (BiFC) assays. NIa-pro interacts with IAAS protein and consequently increasing the stability of IAAS protein. Also, the subcellular localization of both NIa-pro and IAAS protein in the nucleus and cytosol was demonstrated. By converting free IAA (active form) to conjugated IAA (inactive form), IAAS plays a crucial regulatory role in auxin signaling. Transient silencing of IAAS in *N. benthamiana* plants reduced the PVY-mediated symptom induction and virus accumulation. Conversely, overexpression of IAAS enhanced symptom induction and virus accumulation in infected plants. In addition, the expression of auxin-responsive genes was found to be downregulated during PVY infection. Our findings demonstrate that PVY NIa-pro protein potentially promotes disease development via modulating auxin homeostasis.

## Introduction

Plants employ both innate and induced defense responses to offset the attacks by pathogens. Induced defense responses of plants are broadly classified as pathogen-associated molecular pattern (PAMP)-triggered immunity (PTI), and effector-triggered immunity (ETI). Bacterial flagellins and polysaccharides are well-known examples of PAMPs. Pathogen-secreted effector proteins suppress the basal resistance provided by PTI. Plants have evolved resistance (R) proteins to recognize effectors and induce ETI (Jones and Dangl, 2006), which is much stronger than PTI. Salicylic acid (SA) and jasmonic acid (JA) are two major phytohormones associated with PTI and ETI, primarily acting against biotrophic and necrotrophic pathogens, respectively (Alfano and Collmer, 1996; Jones and Dangl, 2006). Other plant hormones, including auxins are also important players in plant disease resistance.

Auxins regulate multiple physiological, growth and developmental processes such as cell elongation and division, shoot and root architecture, root induction, epinasty, and tropic responses (Kieffer et al., 2010). Auxin is also involved in plant responses to abiotic and biotic stress. The presence of auxin is sensed by a combinatorial F-Box TRANSPORT INHIBITOR RESPONSE1/AUXIN SIGNALING F-BOX (TIR1/AFB) family and Aux/IAA co-receptor system (Calderón Villalobos et al., 2012). Binding of auxin with its co-receptor proteins, TIR1/AFB and Aux/IAA proteins, facilitates ubiquitination and degradation of Aux/IAA proteins and leads to transcription of auxin-responsive genes, including auxin-responsive factor (ARF) (Kieffer et al., 2010; Leyser, 2017). The well-studied Indole-3-acetic acid (IAA) is the most predominant form of auxins found in plants. The amount of free IAA (active form) in any specific tissue is tightly regulated by several pathways including synthesis, transport, modification, and degradation. Conjugation of free IAA with amino acids results in either storage conjugate (IAA-Ala and IAA-Leu) or catabolism conjugate (IAA-Asp and IAA-Glu) of IAA (Ludwig-Müller, 2011). Pathogens may promote auxin biosynthesis which modulates plant defense response via rapidly inducing the expression of three groups of genes, the *Aux/IAA* family, the *GRETCHEN HAGEN 3* (*GH3*) family, and the *SMALL AUXIN-UP RNAs* (*SAURs*) (Yamada, 1993; Woodward and Bartel, 2005). The acyl acid amido synthetase activity of GH3 proteins facilitates the conjugation of auxins and JAs, mostly with amino acids. *GH3.5* encodes *Indole-3-acetic acid-amido synthetase* (IAAS), an enzyme that conjugates biologically active free IAA to IAA-Asp. Bacterial and fungal plant pathogens usurp the auxin metabolism pathway of the host, increasing the accumulation of conjugated form, IAA-Asp, and promoting disease development (González-Lamothe et al., 2012).

Auxin signaling plays critical role in almost every biological process of the plant. ARFs are important transcriptional regulators that mediate auxin signaling. However, the role of auxin signaling in plant-virus interaction is understudied (Zhang et al., 2020). Most plant viruses disrupt auxin signaling either by changing Aux/IAA by stabilization of Aux/IAA or by interfering with transcriptional activity of ARFs (Zhang et al., 2020; Müllender et al., 2021). Tobacco mosaic virus (TMV)-encoded 126 kDa replication-associated protein was shown to interact with a subset of Aux/IAA transcriptional regulators (Padmanabhan et al., 2008). More recently, it was shown that disruption of Aux/IAA localization to the nucleus in phloem cells was correlated with greater vascular-loading (i.e. systemic spread) of TMV(Collum et al., 2016). Recent reports had shown that proteins of different plant viruses have evolved to interfere with auxin signaling by interfering with ARFs. Proteins encoded by plant RNA viruses such as rice black-streaked dwarf virus (RBSDV), southern rice black-streaked dwarf virus (SRBSDV), rice stripe virus (RSV), tomato chlorosis virus (ToCV), rice dwarf virus (RDV) disrupt the auxin signaling by inhibiting the transcription activity of ARFs (Jin et al., 2016; Qin et al., 2020; Zhang et al., 2020; Liu et al., 2021). Several genes related to auxin signaling were found to be altered during beet necrotic yellow vein virus infection in sugar beet (Fernando Gil et al., 2020). A tolerant reaction of potato against PVY induces *miR160-ARF10/ARF17* expression that is associated with of *ARF10* and *ARF17* mRNA (Stare et al., 2019).

Potato virus Y (PVY) (genus *Potyvirus*, family *Potyviridae*) is one of the most economically important plant viruses (Gadhave et al., 2020). PVY contains a positive-sense, single-stranded RNA genome of about 9.7 kb. The PVY genome encodes a single polyprotein that is post-translationally cleaved to P1-pro, HC-pro, P3, 6K1, CI, 6K2, VPg, NIa-pro, NIb, and CP (Dougherty and Carrington, 1988). HC-pro serves several functions in potyviral life cycle as a helper component, protease, and silencing suppressor. HC-pro is well-studied among potyvirus proteins and is known to play multifaceted role during viral pathogenesis. HC-pro interacts with various proteasome proteins (PAE1, PAE2, PAA, PBB, and PBE) and interferes with proteasome protease activity (Jin et al., 2007).

Understanding the PVY virus-host interactions could provide important insights into the underlying mechanistic details. Multiple studies have shown that particular host-virus protein-protein interactions either facilitates viral pathogenesis or correlates with antiviral resistance (Bhat et al., 2013; De Ronde et al., 2014; Hallwass et al., 2014; Wang, 2015; Zhao et al., 2016; Garcia-Ruiz, 2019; Gnanasekaran et al., 2019; Ray and Casteel, 2022). However, minimal information is available on the proteins targeted by PVY.

Potyvirus NIa protein consists of two domains, which are cleaved into VPg and NIa-pro by an inefficiently utilized self-proteolytic site. Several putative host proteins were found to interact with NIa-pro protein coded by TEV in *Arabidopsis thaliana* by using affinity purification-mass spectroscopy (Schaad et al., 2000). NIa-pro protein of TEV interacts with eIF4E in tomato and tobacco. This interaction is strain-specific: eIF4E interacts with the NIa-pro protein from the TEV-HAT strain but not with that of TEV-Oxnard strain. However, little information is available about the PVY NIa-pro-host interactome.

PVY reduces the yield of three major crops that contribute to food and nutritional security, potato, pepper, and tomato. PVY exists as biologically distinct strains that differ in the symptoms they cause on potato foliage and tubers, and new strains continue to emerge, mostly through the process of recombination (Lorenzen et al., 2006; Gray, 2007). The relative prevalence of these strains continue to shift and two tuber necrosis causing recombinant strains, PVY^NTN^ and PVY^N-Wi^ are considered to be the most prevalent in the US (Tran et al., 2022). PVY° induces recognizable mosaic symptoms on the leaves of most infected hosts, whereas the necrotic strains PVY^N-Wi^ and PVY^N^ induce milder mosaic symptoms on leaves, but cause necrotic ringspot disease in potato tubers (Gray, 2007). In Europe, PVY^NTN^ has completely displaced PVY° within the past 20 years (Blanchard et al., 2008; Kamangar et al., 2014). PVY° incidence is also decreasing in the U.S. with a dramatic rise in PVY^N-Wi^ and PVY^NTN^ recombinants in the past decade. Interestingly, PVY^NTN^ and PVY^N-Wi^ tend to induce less severe foliar symptoms than PVY° on most cultivars. The milder foliar symptoms induced by PVY^NTN^ and PVY^N-Wi^ may contribute to their rise in incidence since infections may go unnoticed during field inspections conducted for seed certification (Green et al., 2017; Gadhave et al., 2020). The PVY isolate (PVY^NTN^) used in this study was sequenced from the diseased potato plants grown in a commercial field in Washington State, USA.

In this study, we used *Nicotiana benthamiana* and PVY^NTN^ as a model system to investigate plant-PVY interactions. We demonstrate that potato virus Y (PVY)-coded NIa-pro interacts with IAAS and that the modulation of IAAS expression affects the PVY-induced disease development. Also, we show that NIa-pro interacts with and stabilizes the IAAS protein. Based on our findings, we propose a model demonstrating for the role of auxin homeostasis in PVY pathogenicity.

## Materials and methods

### Cloning and production of recombinant plasmid constructs

A naturally occurring PVY^NTN^ strain isolated from infected potato plants grown in a commercial field in Washington State was used. The virus was maintained on *N. benthamiana* plants. The complete RNA genome was obtained as cDNA by RT-PCR using six overlapping primer pairs (PVY1FP/PVY1RP, PVY2FP/PVY2RP, PVY3FP/PVY3RP, PVY4FP/PVY4RP, PVY5FP/PVY5RP, PVY6FP/PVY6RP) (**Supplementary Table 1**). Overlapping amplicons were separately cloned into pJET1.2 vector and sequenced and the complete genome of PVY^NTN^ was deposited (GenBank accession no. ON866943).

To generate the entry clone, pDONR201-NIa-pro, the coding sequence of NIa-pro of PVY^NTN^ was PCR amplified using a primer pair NIaProFP/NIaProRP (**Supplementary Table 1**), and was cloned into the entry vector, pDONR201. To generate pDONR201-IAAS construct, the full-length coding sequence of IAAS (GenBank accession no. ON866944) was PCR-amplified from the cDNA of *N. benthamiana* leaves by using the specific primer pair (IAASFP/IAASRP) and cloned into the pDONR201 vector. The pGBKT7-NIa-pro and pSITE-nEYFP-N1:NIa-pro constructs were generated by mobilizing the NIa-pro from pDONR201-NIa-pro to the pGBKT7-GW and pSITE-nEYFP-N1 vector, respectively. This *in vitro* recombination-mediated mobilization from an entry clone to the destination vector was performed by using Gateway™ LR Clonase™ enzyme mix (Thermo Fisher Scientific) by following the manufacturer’s instructions. Similarly, pGADT7-IAAS, pSITE-nEYFP-C1:IAAS, and pEARLEY103-IAAS constructs were generated by mobilizing the IAAS coding sequence from pDONR201-IAAS to pGADT7-GW, pSITE-nEYFP-C1, and pEARLEY103-GW vectors, respectively. To generate the pTRV-*IAAS*-silencing construct, *IAAS*-fragment (346 bp) was amplified using the primer pair SiIAASFP/SiIAASRP and cloned in antisense orientation in the pTRV2-GW vector. Similarly, to generate the pTRV-*NbPDS*-silencing construct, the fragment of *N. benthamiana Phytoene desaturase* (*NbPDS*) (491 bp) was amplified from the cDNA of *N. benthamiana* leaves by using the specific primer pair SiPDSFP/SiPDSRP (**Supplementary Table 1**) and cloned in antisense orientation in pTRV2-GW vector. To generate the pGR106-IAAS expression construct, the full-length coding sequence of IAAS was amplified with the primer pair PExIAASFP/PExIAASRP, cloned into pJET.12 and subcloned in *Cla1* and *Sal1* restriction site of the pGR106 vector. Sequences of all the primers used in this study are listed in **Supplementary Table 1**.

### Yeast two-hybrid library screening

The pGBKT7-NIa-pro construct containing the coding sequence of NIa-pro of PVY^NTN^ in-frame with the yeast GAL4 binding domain protein was used as a bait construct for the Y2H library screening. The mRNA was isolated from leaves of the *N. benthamiana* plants using Oligotex mRNA Mini Kit (Qiagen) and by following the manufacturer’s instructions. The *N. benthamiana* Y2H cDNA library was constructed using mRNA isolated from the leaves of the *N. benthamiana* plants and Matchmaker^®^ Gold Yeast Two-Hybrid System kit (Takara). Construction of *N. benthamiana* Y2H cDNA library and screening were performed as illustrated in the Matchmaker Gold Yeast Two-Hybrid System User Manual (Takara). The *N. benthamiana* Y2H library was screened using pGBKT7–NIa-pro as the bait in *Saccharomyces cerevisiae* strain Y2HGold. Yeast colonies containing potential interacting partners of NIa-pro were selected based on the ability of the co-transformed cells to grow on synthetic media containing aureobasidin and lacking Trp and Leu. Subsequently, those yeast cells were screened with high stringency and were selected based on their ability to grow on synthetic media lacking Trp, Leu, and His and containing 1mM 3-amino-1,2,4-triazole. The prey plasmids present in the yeast colonies that were able to grow on synthetic media lacking Trp, Leu, and His and containing 3-amino-1,2,4-triazole were rescued and sequenced.

### Yeast two-hybrid assay

Y2H assays were performed by co-transforming *S. cerevisiae* strain Y2HGold cells with plasmid pGBKT7-NIa-pro plus pGADT7-IAAS. A pGBKT7-P53 and pGADT7-TAg plasmid combination was used as positive control, while a pGBKT7 and pGADT7 vector combination was used as the negative control. The interaction was tested by growing the yeast co-transformants on synthetic screening media lacking Leu, Trp, and His and containing 1 mM 3-amino-1,2,4-triazole and X-α-Gal at 30°C for 4 days (Gnanasekaran et al., 2019; Gnanasekaran et al., 2023).

### Bimolecular fluorescence complementation assay

BiFC assays were performed as described previously (Gnanasekaran et al., 2019). Leaves of three-week old *N. benthamiana* plants were co-infiltrated with *Agrobacterium tumefaciens* strain GV3101 cells containing pSITE-nEYFP-N1: NIa-pro and pSITE-nEYFP-C1: IAAS. Similarly, *Agrobacterium* cells containing pSITE-nEYFP-N1 and pSITE-nEYFP-C1, pSITE-nEYFP-N1: NIa-pro and pSITE-nEYFP-C1, pSITE-nEYFP-N1 and pSITE-nEYFP-C1: IAAS negative combinations were infiltrated into leaves of *N. benthamiana* plants. Forty eight hours post-infiltration, the abaxial epidermal cells of the infiltrated *N. benthamiana* leaves were examined using a Leica TCS SP8 X confocal laser-scanning microscope using 20X objective lens, and the LAS X software (Leica Microsystems, Wetzlar, Germany). The nuclei of the epidermal cells of the infiltrated leaves were stained with 4, 6-diamidino-2-phenylindole (DAPI) (Gnanasekaran et al., 2019). For detection of DAPI fluorescence, 405 nm blue diode was used for excitation and the emitted light was collected between 435 to 485 nm. For detecting reconstituted yellow fluorescent protein (YFP), 514 nm excitation was used and the emitted light was collected between 530 nm to 570 nm.

### Subcellular localization by fluorescent confocal microscopy

Subcellular localization of the NIa-pro and *N. benthamiana* IAAS protein was performed as described previously (Bhattacharyya et al., 2015). The leaves of three-week old *N. benthamiana* plants were infiltrated with *A. tumefaciens* strain GV3101 cells containing localization constructs (Bhattacharyya et al., 2015) namely, pCAMBIA1302 vector, pCAMBIA1302-NIa-pro, pEARLEY103-IAAS. The nuclei of the epidermal cells were stained with DAPI. After 48 hours post-infiltration, the abaxial epidermal cells of the infiltrated *N. benthamiana* leaves were examined using a Leica TCS SP8 X confocal laser-scanning microscope, and the LAS X software (Leica Microsystems, Wetzlar, Germany). For detection of DAPI fluorescence, 405 nm blue diode was used for excitation and the emitted light was collected between 435 and 485 nm. For green fluorescent protein (GFP) fluorescence, 488 nm excitation was used and the emitted light was collected between 515 nm to 545 nm.

### Tobacco rattle virus-based silencing of endogenous *Indole-3-acetic acid-amido synthetase* in *Nicotiana benthamiana* plants

Transient silencing was carried out using TRV-based gene silencing vectors. pTRV1 (CD3-1039) and pTRV2-GW (CD3-1041) vectors (Liu et al., 2002) were obtained from the Arabidopsis Biological Resource Center, OH, United States. pTRV1, pTRV2, pTRV2-*IAAS*, and pTRV2-NbPDS silencing constructs were transformed into *A. tumefaciens* strain EHA105. To achieve silencing of endogenous *IAAS*, two fully expanded top leaves of three-week old *N. benthamiana* plants were co-infiltrated with *Agrobacterium* cells carrying pTRV1 and pTRV2-*IAAS*. Plants with co-infiltrated *Agrobacterium* carrying either pTRV1 and pTRV2-NbPDS, or pTRV1 and pTRV2 combination served as positive and negative controls, respectively. The infiltrated plants were grown at 22**°**C, 70% relative humidity, and in 16h of light/8h of dark photoperiod with light intensity of 100 µmol/m^2^/s.

### Transient overexpression of IAAS in *Nicotiana benthamiana plants*

A modified potato virus X (PVX) vector, pGR106 (Lu et al., 2003; Gupta et al., 2022) was used for transient overexpression of IAAS in *N. benthamiana* plants. The pGR106 vector and pGR106-IAAS overexpression construct were mobilized into *A. tumefaciens* strain GV3101 containing helper plasmid, pJIC SA_Rep (Gupta et al., 2022). Overexpression of IAAS was accomplished by infiltrating the lower leaves of three week-old *N. benthamiana* plants with *Agrobacterium* cells carrying pGR106-IAAS. Plants infiltrated with *Agrobacterium* carrying pGR106 vector served as the control. The infiltrated plants were grown at 22**°**C, 70% relative humidity, and in 16h of light/8h of dark photoperiod with light intensity of 100 µmol/m^2^/s.

### Plant inoculations

For PVY inoculation, leaves of the *N. tabacum* plants previously infected with PVY^NTN^ were used as source of inoculum. One gram of infected tobacco leaves was ground in 10 ml of 100 mM sodium phosphate buffer (pH 7.2) containing 0.4% (v/v) β-mercaptoethanol, and 1:10 (wt/vol) of carborundum (Manasseh et al., 2022). The sap extracted from PVY-infected leaf tissue was applied by manually rubbing the inoculum on the adaxial side of the leaves of *N. benthamiana* plants. While sap of healthy tobacco plants was used for mock-inoculation. PVY symptom severity were visually scored on a scale of 0-4 (0=no symptoms, 1= mild leaf curling and/or mild mosaic, 2= severe leaf curling and/or typical chlorosis, 3=mild necrosis and/or stunted growth, 4=severe necrosis). Symptom severity graphs were plotted using the score recorded from atleast 9 plants for each treatment and by using the sigma plot software.

### Enzyme-linked immunosorbent assay

The relative amount of the viral load present in the systemically infected top leaves of the infected plants was analyzed by Double-antibody Sandwich (DAS) Enzyme-Linked Immunosorbent Assay (ELISA). DAS-ELISA was performed using the ELISA reagent set (SRA 20001, Agdia, IN, USA). One gram of *N. benthamiana* leaf sample was ground in general extract buffer (GEB) (Agdia, Inc.). Leaf sap (100 µl) was used for measuring the viral titer using DAS-ELISA, with two technical replicates and three biological replicates for each treatment (Manasseh et al., 2022).

### Total RNA extraction and quantitative reverse transcription-PCR

For each treatment, top two leaves from atleast three *N. benthamiana* plants were collected. Total RNAs from the collected *N. benthamiana* leaf tissues were extracted using RNeasy Plant Mini Kit (Qiagen, St. Louis, MO, USA) and were treated with DNase I (Sigma, St. Louis, MO, USA). The cDNA synthesis was carried out using random hexamers and MLV reverse transcriptase (Fermentas, USA). The RT-qPCR analysis was carried out as described previously (Zhai et al., 2019) with slight modifications. *Protein phosphatase 2A* (PP2A) was used as an internal reference gene (Cassan-Wang et al., 2012). RT-qPCR analysis performed in this study was carried out with three biological and three technical replicates. Statistical significance was determined by one-way ANOVA. Sequences of the primers used in this study are listed in **Supplementary Table 1**.

### Immunoblotting analysis

Leaves of three- to four-week-old *N. benthamiana* plants were co-infiltrated with *Agrobacterium* cells carrying constructs that express either IAAS-GFP and N-terminal of YFP (YFP-N1) or IAAS-GFP and YFP-N1-NIa-pro. After 6 dpi, total protein from the infiltrated leaves was extracted in protein extraction buffer containing 1X phosphate buffer (pH 7.4), 5 mM β-mercaptoethanol, 1 mM Phenylmethylsulfonyl fluoride, and plant protease inhibitor cocktail (P9599, Sigma-Aldrich, USA). Equal amounts of total protein was analyzed by sodium dodecyl sulfate-polyacrylamide gel electrophoresis (SDS-PAGE) and immunoblotting assay using an anti-GST-HRP antibody (sc-9996 HRP, Santa Cruz Biotechnology, TX, USA).

## Results

### Infectivity and molecular characterization of PVY strain

**Figure 1:**
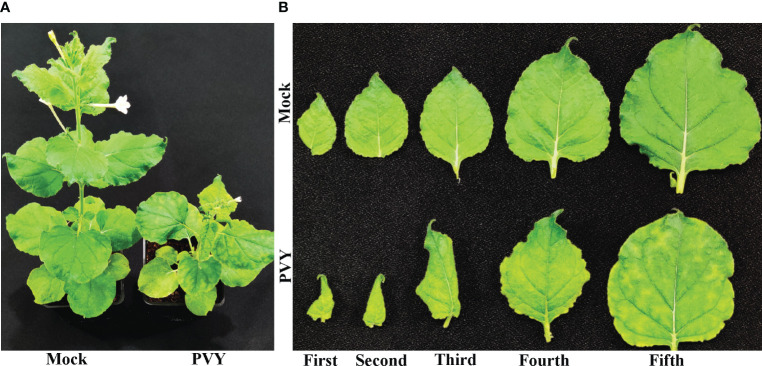
Potato virus Y tuber necrotic strain (PVY^NTN^)-mediated symptom induction on *Nicotiana benthamiana*. **(A)**. Mock- or potato virus Y tuber necrotic strain (PVY^NTN^)-inoculated *N. benthamiana* plants and **(B)** first to fifth leaves from the top at 15 days post-inoculation.

*N. benthamiana* plants that were mechanically inoculated with sap containing PVY^NTN^ developed mild leaf curl symptoms at around 9 days post-inoculation (dpi) on systemically infected top leaves. Later at 15 dpi, plants showed severe chlorotic mosaic, leaf curling affecting the leaf lamina, and stunted growth, while the mock-inoculated *N. benthamiana* plants remained symptomless (**Figures 1A, B**). Similarly, *N. tabacum* plants inoculated with PVY^NTN^, beginning at 15 dpi, showed severe veinal chlorosis affecting the mid-veins and later developed severe chlorotic mosaic at 20 dpi, while the mock-inoculated *N. tabacum* plants remained symptomless (**Figure S1**). These observations suggested that PVY^NTN^ strain used in this study could induces a recognizable chlorotic mosaic symptoms on the leaves of both *N. benthamiana* and *N. tabacum* plants. The complete genome of PVY^NTN^ isolate used in this study was deposited in GenBank database (GenBank accession no. ON866943). The sequence showed a nucleotide identity of 99.79% with PVY^NTN^ isolate HR1 (GenBank accession no. FJ204166.1).

### PVY-encoded NIa-pro protein interacts with IAAS in *Nicotiana benthamiana*

**Figure 2:**
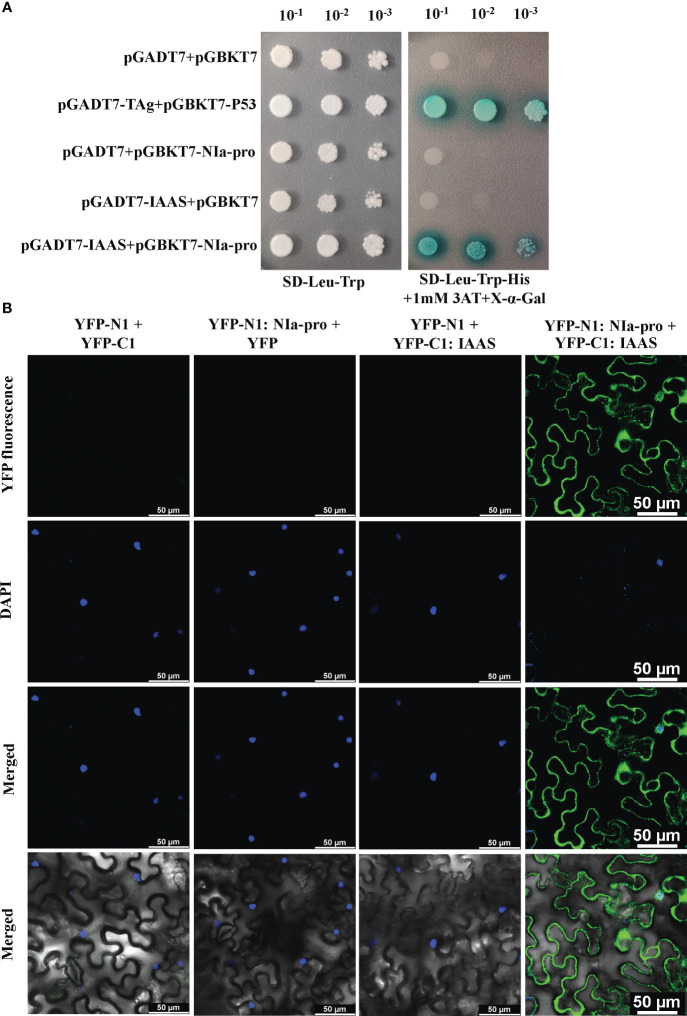
Potato virus Y-encoded NIa-pro protein interacts with *Nicotiana benthamiana Indole-3-acetic acid-amido synthetase* (IAAS). **(A)**. Yeast strain Y2H-Gold cells were co‐transformed with different combinations of yeast two-hybrid assay constructs as shown. The co‐transformed Y2H-Gold cells were grown on either synthetic dropout medium lacking Leu and Trp (SD-Leu-Trp) or selection medium lacking Leu, Trp, and His (SD-Leu-Trp-His) supplemented with 1 mM 3‐amino‐1,2,4‐triazole (3AT) plus X-α-Gal as serial dilution from cultures OD_600_ of 1.0. Y2H-Gold cells co-transformed with pGADT7-TAg and pGBKT7-P53 or pGADT7 and pGBKT7 act as positive and negative controls, respectively. **(B)**. Different combinations of *Agrobacterium* cells harboring bimolecular fluorescence complementation assay constructs were co‐infiltrated into the leaves of 3- to 4-week-old *N. benthamiana* plants. After 48 hours the nuclei were stained with 4, 6-diamidino-2-phenylindole (DAPI) and abaxial epidermal leaf cells were visualized under a confocal microscope. The reconstituted yellow fluorescent protein (YFP) fluorescence was observed in the leaves co-infiltrated with YFP-N1:NIa-pro and YFP-C1:IAAS. The leaves co-infiltrated with either YFP-N1 plus YFP-C1, YFP-N1:NIa-pro plus YFP-C1, or YFP-N1 plus YFP-C1:IAAS act as negative controls. Images in rows 1, 2, 3, and 4 are showing YFP fluorescence image, DAPI fluorescence image, merged YFP and DAPI fluorescence image, and merged brightfield with YFP and DAPI fluorescence image. Scale bar represents 50 µM.

To explore the role of PVY-encoded NIa-pro protein during host-virus interaction, Y2H library was screened by using pGBKT7-NIa-pro as bait and *N. benthamiana* Y2H cDNA library. The *N. benthamiana* IAAS was found to be one of the potential host-interacting partners of NIa-pro protein. The interaction between the NIa-pro protein and *N. benthamiana* full-length IAAS protein was confirmed by Y2H assay. Yeast transformants carrying BD-NIa-pro and AD-IAAS plasmids were able to grow on selection plates deficient in Leu, Trp, and His and supplemented with 1 mM 3-amino-1,2,4-triazole and X-α-Gal (**Figure 2A**). The yeast transformants carrying positive control plasmids (AD-T_Ag_ and BD-P_53_) grew on the selection plates, while yeast transformants with negative control plasmids (AD and BD, AD-IAAS and BD or AD and BD-NIa-pro) were unable to grow (**Figure 2A**). These results confirmed the interaction of NIa-pro protein with the IAAS protein in yeast cells.

Further, BiFC assays were carried out in *N. benthamiana* plants using the BiFC vectors, pSITE-nEYFP-N1 and pSITE-nEYFP-C1. The nuclei of the epidermal cells were stained using DAPI. YFP fluorescence was observed in the epidermal cells of the leaves co-infiltrated with *Agrobacterium* cells containing pSITE-nEYFP-N1:NIa-pro and pSITE-nEYFP-C1:IAAS constructs (**Figure 2B**; **Figure S2**). This YFP signal was detected predominantly in the cytoplasm and some in the nucleus and/or perinuclear regions. However, no YFP signal was observed in the epidermal cells of the leaves co-infiltrated with pSITE-nEYFP-N1 and pSITE-nEYFP-C1, pSITE-nEYFP-N1:NIa-pro and pSITE-nEYFP-C1, or pSITE-nEYFP-N1 and pSITE-nEYFP-C1:IAAS combinations (**Figure 2B**). These results demonstrate that NIa-pro protein interacts with IAAS protein.

### Subcellular localization of NIa-pro and IAAS protein

The results of our BiFC experiment demonstrated that NIa-pro interacts with IAAS predominantly in the cytosol (**Figure 2B**). NIa-pro protein of PVY and TuMV, was shown to localize to the nucleus and/or vacuoles (Bak et al., 2017). To better understand the role of NIa-pro protein in the host-virus interaction, we performed subcellular localization assays. NIa-pro-GFP fluorescence was observed at the nuclei (co-localized with signal from DAPI), cytosol, and nuclear inclusion (**Figure S3**). IAAS-GFP fluorescence also was observed in the cytosol, as well as the perinuclear and nuclear region, whereas fluorescence of the free GFP (control) was observed throughout the whole cell (**Figure S3**), suggesting that both NIa-pro and IAAS independently localize to the cytosol and to some degree to the nucleus and/or perinuclear regions.

### Silencing of the *IAAS* gene reduces PVY pathogenesis in *Nicotiana benthamiana*

Silencing of the *IAAS* gene that encodes the IAAS protein was achieved in *N. benthamiana* plants by infiltrating *Agrobacterium* cells containing the pTRV-*IAAS* silencing construct, while the pTRV vector infiltrated plants served as the control for silencing experiments. The pTRV-*NbPDS* construct infiltrated *N. benthamiana* plants served as phenotypic control for gene silencing and showed photobleaching at10 dpi; (**Figure S4A**). The pTRV-*IAAS*-infiltrated *N. benthamiana* plants showed around 5 cm increased height compared to the pTRV- infiltrated plants (**Figure S4B**). *IAAS* transcript levels in the upper leaves of pTRV-*IAAS* or pTRV- infiltrated plants at 10 dpi were measured by semiquantitative RT-PCR. Silencing of the *IAAS* gene was reflective of the reduction in *IAAS* mRNA level in pTRV-*IAAS* infiltrated plants compared to pTRV- plants (**Figure S5**).

**Figure 3:**
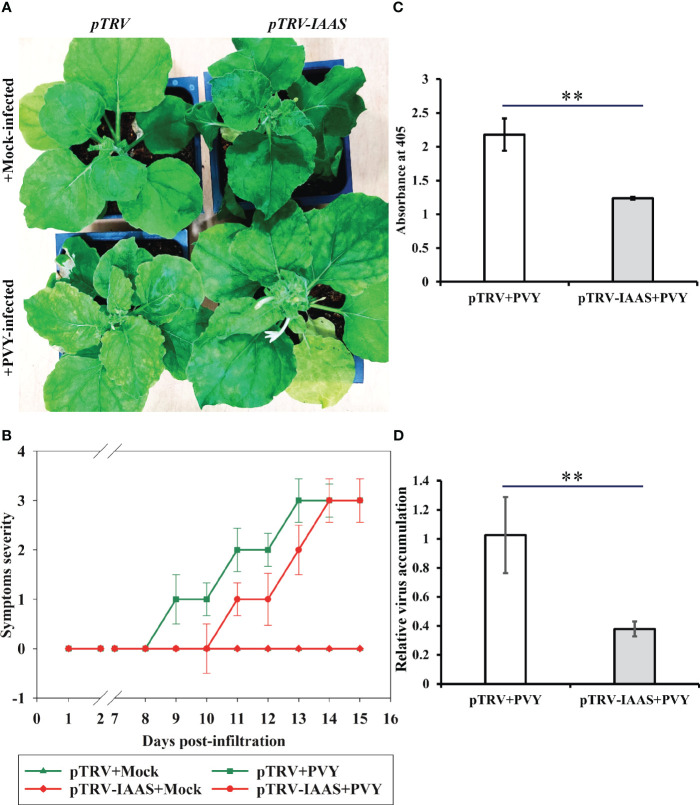
Silencing of *Indole-3-acetic acid-amido synthetase* decreases potato virus Y-mediated disease development in *Nicotiana benthamiana*. **(A)** Potato virus Y (PVY) induced symptoms on *Indole-3-acetic acid-amido synthetase* (*IAAS*)-silenced or pTRV-infiltrated *N. benthamiana* plants at 15 days post-inoculation (dpi). **(B)** Appearance of PVY induced symptoms at different dpi on *IAAS*-silenced or pTRV-infiltrated *N. benthamiana* plants. Infected *N. benthamiana* plants were visually scored for PVY symptoms on a scale of 0-4 (0=no symptoms, 1= mild leaf curling and/or mild mosaic, 2= severe leaf curling and/or typical chlorosis, 3=mild necrosis and/or stunted growth, 4=severe necrosis). This symptom severity graph was plotted using the Sigma plot 14.5 which rounds mean values to the nearest whole number in the severity ranking score. Error bar represents standard deviation. Comparative level of PVY viral-titer **(C)** and viral RNA **(D)** in *IAAS*-silenced or pTRV-infiltrated control plants tested by double antibody sandwich ELISA and quantitative real-time PCR, respectively. ** indicates P-value < 0.01 as determined by one-way ANOVA.

To investigate the role of IAAS protein in PVY pathogenesis, *IAAS*-silenced plants and the pTRV-control *N. benthamiana* plants were inoculated with PVY^NTN^ isolate. PVY-inoculated pTRV control plants displayed typical symptoms such as leaf curling, severe chlorotic mosaic, and stunted growth, while PVY-inoculated *IAAS* silenced plants showed leaf curling and chlorotic mosaic but not stunted growth at 15 dpi (**Figure 3A**). PVY-infected pTRV control plants showed PVY symptoms at around 9 dpi, whereas PVY infected *IAAS*-silenced had showed delayed symptoms at around 11 dpi (**Figure 3B**). The accumulation of PVY virus-particle and viral RNA were assessed by DAS-ELISA and RT-qPCR, respectively. At 15 dpi, the accumulation of PVY in the upper leaves of PVY-infected *IAAS* silenced plants was found to be reduced compared to that in PVY-infected pTRV-control plants (**Figure 3C**). Similarly, *IAAS* silenced plants showed decreased PVY RNA level compared to that of pTRV-control plants (**Figure 3D**). Thus, silencing of *IAAS* reduced the susceptibility of *N. benthamiana* plants to PVY. Taken together, these results suggested that silencing of *IAAS* gene probably increases the level of free auxin and contributes to both promoting plant growth and reduced susceptibility of PVY in the *N. benthamiana* plants.

### Transient overexpression of IAAS protein enhances the PVY pathogenesis in *Nicotiana benthamiana*

Transient overexpression of IAAS protein was accomplished in *N. benthamiana* plants using the pGR106-IAAS construct, while pGR106-infiltrated plants served as control. To determine the role of IAAS protein during PVY infection, we first analyzed plants infiltrated with pGR106-IAAS or pGR106-vectors without PVY challenge. The pGR106 vector, being a PVX-based plant expression vector, the mock-treated pGR106-IAAS- and pGR106-infiltrated plants showed mild symptoms of PVX. IAAS transcripts levels in upper leaves of pGR106-IAAS or pGR106-control infiltrated plants at 7 dpi were measured by semiquantitative RT-PCR. Transient overexpression of the *IAAS* gene was confirmed as an increased accumulation of *IAAS* mRNA in the pGR106-IAAS infiltrated plants as compared to pGR106-control plants (**Figure S5**).

**Figure 4:**
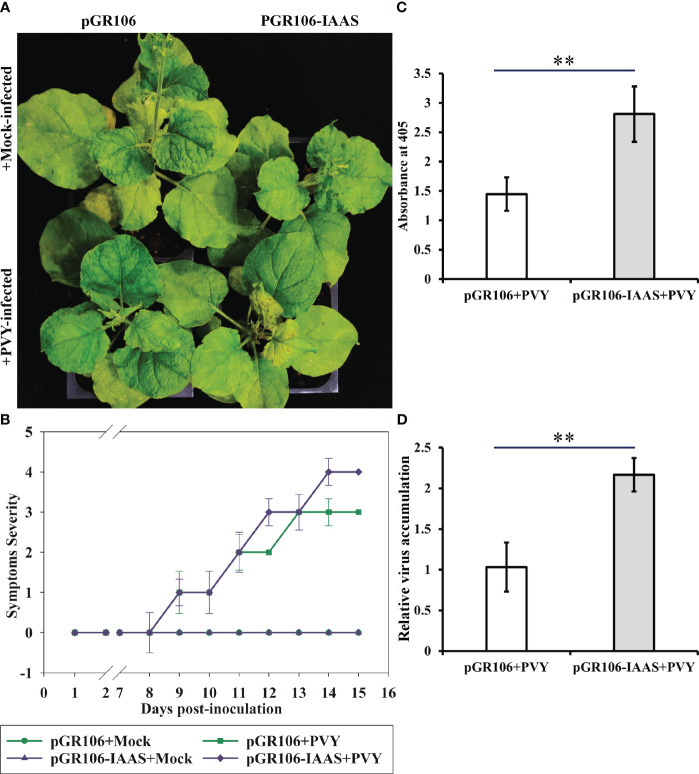
Overexpression of Indole-3-acetic acid-amido synthetase aggravates potato virus Y-mediated symptoms and increase the virus accumulation. **(A)** Potato virus Y (PVY) induced symptoms on Indole-3-acetic acid-amido synthetase (IAAS)-overexpressing or pGR106-infiltrated *Nicotiana benthamiana* plants at 15 days post-inoculation (dpi). **(B)** Appearance of PVY induced symptoms at different dpi on IAAS-overexpressing or pGR106-infiltrated *N. benthamiana* plants. Infected *N. benthamiana* plants were visually scored for PVY symptoms on a scale of 0-4 (0=no symptoms, 1= mild leaf curling and/or mild mosaic, 2= severe leaf curling and/or typical chlorosis, 3=mild necrosis and/or stunted growth, 4=severe necrosis). This symptom severity graph was plotted using the Sigma plot 14.5 which rounds mean values to the nearest whole number in the severity ranking score. Error bar represents standard deviation. Comparative level of PVY virus particle **(C)** and viral RNA accumulation **(D)** in IAAS-overexpressing or pGR106-infiltrated control plants tested by double antibody sandwich ELISA and quantitative real-time PCR, respectively. ** indicates P-value < 0.01 as determined by one-way ANOVA.

Later, to study the role of IAAS protein during the PVY pathogenesis, we inoculated IAAS-overexpressing plants and the pGR106-control *N. benthamiana* plants with PVY^NTN^ isolate. The PVY-mediated symptom induction was found to be enhanced in pGR106-IAAS-infiltrated *N. benthamiana* plants compared to pGR106-infiltrated plants (**Figure 4A**). The pGR106-IAAS-infiltrated *N. benthamiana* plants showed necrotic spots on leaves around 13 dpi (**Figure 4A**; **Figure S6**). PVY-infected IAAS overexpressing plants showed exacerbated PVY symptoms compared to that of pGR106-infiltrated control plants (**Figures 4A, B**) during later stage of infection (after 13 dpi). The accumulation of PVY virus particles and viral RNA was accessed by DAS-ELISA and RT-qPCR, respectively. At 15 dpi, the accumulation of PVY virus particles in the upper leaves of PVY-infected pGR106-IAAS-infiltrated plants was found to be significantly higher than the PVY-infected pGR106-infiltrated plants (**Figure 4C**). Similarly, IAAS overexpressing plants showed significantly higher PVY RNA level compared to that of pGR106-control plants (**Figure 4D**). These results indicate that overexpression of IAAS increases the susceptibility for PVY in *N. benthamiana* plants.

### NIa-pro stabilizes IAAS protein and downregulates auxin-responsive genes in *Nicotiana benthamiana* plants during PVY infection

**Figure 5:**
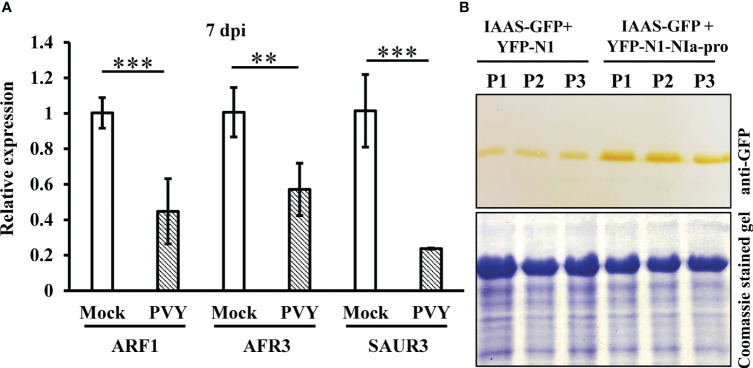
NIa-pro protein stabilizes IAAS protein and downregulates auxin-responsive genes in *N*. *benthamiana* plants during PVY infection. **(A)** Relative expression of selected auxin-responsive genes, *Auxin response factor 1* (ARF1*), Auxin response factor 3* (ARF3), and *small auxin upregulated RNA 3* (SAUR3) in the potato virus Y infected *N. benthamiana* plants at 7 dpi tested by RT-qPCR. ** indicates P-value < 0.01 and *** indicates P-value < 0.001 as determined by one-way ANOVA. **(B)** The leaves of *N. benthamiana* plants were co-infiltrated with *Agrobacterium* cells harboring constructs that expresses either IAAS-GFP and N-terminal of YFP (YFP-N1) or IAAS-GFP and YFP-N1-NIa-pro. An equal amount of total protein extracted from leaves of infiltrated *N. benthamiana* plants were used for SDS-PAGE and immunoblotting analysis. Immunoblotting was carried out using anti-GST HRP antibody. Coomassie‐stained gel served to monitor the equal loading of protein. P1, P2, and P3 indicates samples from three independent plants.

PVY NIa-pro interacts with IAAS protein and modulates PVY-induced symptoms and virus accumulation in *N. benthamiana* plants. To further study the possible importance of NIa-pro-IAAS interaction, we investigated the expression of auxin-responsive genes during PVY infection. For this purpose, *N. benthamiana* plants were inoculated with PVY and were tested for the relative expression of selected auxin-responsive genes, *Auxin response factor 1* (ARF1), *Auxin response factor 3* (ARF3), and *small auxin upregulated RNA 3* (SAUR3) at 24 hpi, 48 hpi, and 7 dpi by RT-qPCR in the systemically infected leaves. Results showed that expression of ARF1, ARF3, and SAUR3 were not significantly altered at 24 hpi and 48 hpi in the PVY-infected *N. benthamiana* plants compared to mock-inoculated plants (**Figure S7**). However, at 7 dpi, expression of all the tested auxin-responsive genes was found to be significantly downregulated in the PVY-infected *N. benthamiana* plants compared to mock-inoculated plants (**Figure 5**).

This observation led us to the hypothesis that during PVY infection its NIa-pro protein interacts with and stabilizes IAAS protein, which would lead to increased conjugated IAA levels and subsequently downregulation of the auxin-responsive gene expression. To test this hypothesis, leaves of *N. benthamiana* plants were co-infiltrated with *Agrobacterium* cells carrying constructs that expresses either IAAS-GFP and N-terminal of YFP (YFP-N1) or IAAS-GFP and YFP-N1-NIa-pro. After six dpi, the level of IAAS-GFP was determined by Western blotting using anti-GFP antibody. At six dpi, IAAS protein accumulation was found to higher in the leaves infiltrated with IAAS-GFP and YFP-N1-NIa-pro expressing constructs compared to those infiltrated with IAAS-GFP and YFP-N1. Taken together, these results suggest that NIa-pro protein interacts with and stabilizes IAAS protein. This probably leads to an increased conjugated IAA level and contributes to downregulation of auxin-responsive genes.

## Discussion

JA and SA are the two phytohormones that play an important role in generation of plant defense response against pathogen attack (Tamaoki et al., 2013). Abscisic acid (ABA), auxin, brassinosteroid (BR), cytokinins (CK), and gibberellins (GA) are also shown to have a role in defense response (Chiang et al., 2015; Ludwig-Müller, 2015). RDV P2 protein interacts with ent-Kaurene oxidases and interfere Gibberellin, GA_1_ production leading to dwarfing symptoms in rice (Zhu et al., 2005). TMV coat protein suppresses the SA-mediated defense response by stabilizing DELLA proteins (Rodriguez et al., 2014).

Plant-virus interaction greatly influences phytohormone production, and homeostasis (Müllender et al., 2021; Pan et al., 2021). An increasing body of evidence suggests that different plant RNA virus-encoded proteins interfere with the auxin signaling as a common pathogenesis strategy. ARF17 provides antiviral defense against plant RNA viruses (Zhang et al., 2020). RBSDV-encoded SP8 protein, SRBSDV-encoded SP8 protein, and rice stripe virus-encoded P2 protein interact with ARF17. Viral protein interaction with ARF17 interferes with its dimerization, DNA binding ability and thereby represses its transcription activation ability (Zhang et al., 2020). Auxin decreases the OsIAA10 protein level and releases a group of OsIAA10-interacting ARFs, which consequently mediates the antiviral defense response. RDV-encoded P2 viral protein subverts this antiviral defense by stabilizing the OsIAA10 protein (Jin et al., 2016; Zhang et al., 2020). In the presence of auxin, the SCF complex–mediated ubiquitin pathway degrades Aux/IAA protein and triggers auxin signaling. ToCV-encoded p22 protein manipulates the SCF complex protein to suppress auxin signaling and promotes viral pathogenesis (Liu et al., 2021). TMV 126 KDa protein interacts with and disrupts Aux/IAA nuclear localization and promotes disease development (Padmanabhan et al., 2008; Collum et al., 2016).

In this study, we have identified that PVY NIa-pro protein interacts with IAAS protein, an enzyme that regulates the level of active auxin and a homolog of GH3.5 gene product in *N. benthamiana*. BiFC assay showed that NIa-pro and IAAS proteins interact predominately in the cytosol. An earlier study in Arabidopsis showed the presence of six genes that specifically conjugate IAA, and one of them was shown to act on both IAA and SA (Staswick et al., 2002). *GH3.5* gene-encoded IAAS conjugates the auxin with aspartate, a process that regulates the level of active IAA.

From the studies on *Botrytis cinerea* and *Pseudomonas syringae*, it was evident that fungal and bacterial plant pathogens promote disease development in Arabidopsis by increasing the accumulation of conjugated-IAA and by interfering with the auxin signaling pathways (González-Lamothe et al., 2012). On the other hand, GH3.8 activates disease resistance against *Xanthomonas oryzae* in rice. *GH3.8* gene-encoded IAA-amino synthetase prevents the accumulation of free IAA. GH3.8-mediated disease resistance is SA signaling- and JA signaling-independent (Ding et al., 2008). However, there have been no reports of manipulation of auxin signaling via modulating auxin homeostasis in case of plant viruses. Findings from this study showed attenuated symptom induction and reduced accumulation of PVY in *IAAS* gene-silenced *N. benthamiana* inferred that increased free IAA levels contribute to an antiviral state. Similarly, exacerbated symptoms and increased PVY accumulation in *N. benthamiana* plants overexpressing the IAAS protein suggesting that an increase in conjugated IAA level promotes susceptibility to PVY. Thus, the free auxin could contribute to resistance against PVY infection.

Regulation of the auxin-responsive genes *ARF1*, *ARF3*, and *SAUR3* during PVY infection, and interaction between PVY- NIa-pro and IAAS proteins, suggested that PVY NIa-pro interacts with and probably stabilizes the IAAS protein. Higher IAAS activity could lead to reduction of IAA levels and transcriptional down regulation of auxin-responsive genes. Our findings support a model wherein NIa-pro subverts the host defense response that is mediated by auxin signaling through targeting and stabilizing the IAAS. NIa-pro’s interaction with IAAS results in a stabilized active IAAS protein that continues to produce conjugated IAA from free auxin and thus down regulates auxin-responsive genes (ARF1, AFR3 and SAUR3). Further work showing the activity of the NIa-pro-IAAS complex could provide additional insights into this interaction.

**Figure 6:**
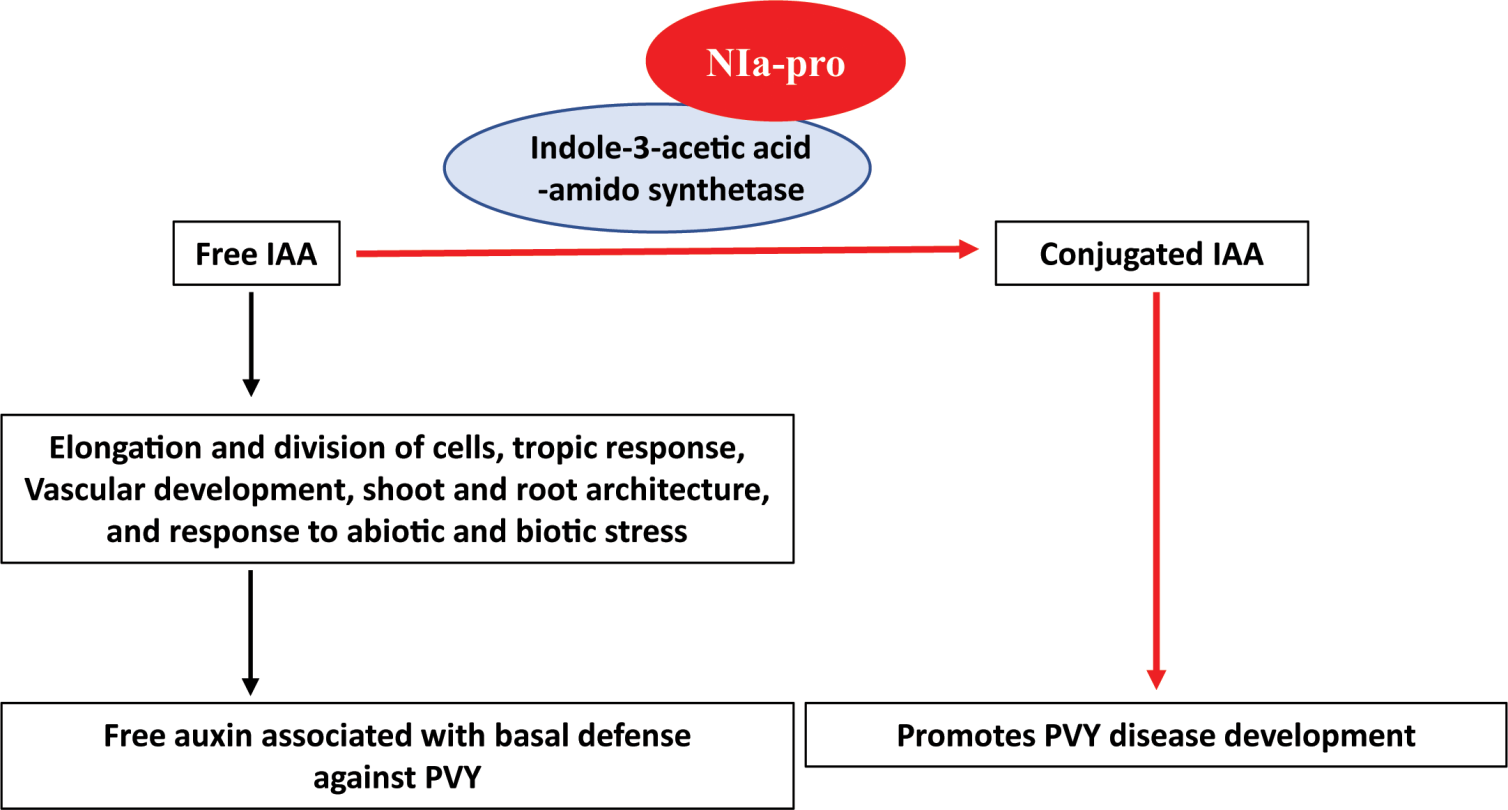
Working model of NIa-pro of potato virus Y - *Indole-3-acetic acid-amido synthetase* interaction. Free IAA (Free auxin) is involved in diverse processes of the plant such as elongation and division of cells, tropic response, vascular development, shoot and root architecture, and response to abiotic and biotic stress. It is associated with basal immunity against plant pathogens. *Indole-3-acetic acid-amido synthetase* (IAAS) is an enzyme that regulate the action of free auxin by conjugating with amino acid, aspartic acid. Potato virus Y (PVY) encoded-NIa-pro protein interacts with IAAS and probably stabilizes the IAAS protein. Consequently, IAAS convert the free IAA to conjugated IAA leading to downregulation of auxin-responsive gene and promotes diseases PVY development.

Based on previous reports and our current findings, we propose a model for NIa-pro-IAAS interaction (**Figure 6**). In this model, free IAA governs diverse processes including basal immunity against plant pathogens. Free IAA is converted into conjugated IAA by IAAS. NIa-pro dependent stabilization of IAAS probably suppresses plant immunity by depleting free auxin. Reduced symptom induction and virus accumulation in the PVY-infected, IAAS-silenced *N. benthamiana* plants suggested that PVY usurps the auxin-mediated defense response by depleting free auxin pool through interaction with IAAS. Exacerbated symptoms and virus accumulation in the PVY-infected, IAAS overexpressing *N. benthamiana* plants suggests that higher abundance of IAAS protein promotes the susceptibility of to PVY. It is likely that during PVY infection, NIa-pro interacts with and stabilizes the IAAS. Subsequently, the IAAS protein with increased stability converts more of the free IAA into conjugated IAA leading to the downregulation of auxin-responsive genes and thereby promoting disease development. The relative levels of free and conjugated auxin in the plants following PVY infection need to be investigated in future. This model suggests a novel strategy employed by a plant virus to promote disease through suppression of auxin signaling. The next steps will focus on detailed characterization of cross-talk between auxin signaling and immunity pathways in the context of interaction between NAi-pro and IAAS, and PVY pathogenicity. 

## Data availability statement

The datasets presented in this study can be found in online repositories. The names of the repository/repositories and accession number(s) can be found in the article/**Supplementary Material**.

## Author contributions

PG, and HP planned and designed the research; PG, HK, YZ performed research; PG, YZ, AS, and HP analyzed and interpreted the data; PG and HP wrote the manuscript.
