# Supplementary material for: A plant virus protein, NIa-pro, interacts with Indole-3-acetic acid-amido synthetase, whose levels positively correlate with disease severity
Source: Front Plant Sci. 2023 Sep 11;14:1112821. doi: 10.3389/fpls.2023.1112821 (PMC10519798; doi:10.3389/fpls.2023.1112821)
Supplement: Supplementary file 1 [file DataSheet_1.docx]

**Supplementary Table-1**: List of primers used in this study.

| **Primer name** | **Sequence** |
| --- | --- |
| PVY1 FP | GTCGACAAATTAAAACAACTCAATACAAC |
| PVY1 RP | GGATCCAGGTTTTTGAAAGGTGATTGTTGC |
| PVY2 FP | GTCGACAATGAAGTATTCAAGTCTATAGG |
| PVY2 RP | GGATCCCCCAAGAATGCTTGTGGTGATAT |
| PVY3 FP | GTCGACGCAGATTTGAAAGGGTTATACAAC |
| PVY3 RP | GGTACCAATTTCTAGGCAACCGTGCTTC |
| PVY4 FP | GTCGACCAAAGGTTGATGGCAGAACAAT |
| PVY4 RP | GGATCCTCTTGGATGTCTCTAATATCAGC |
| PVY5 FP | GTCGACGAGCTCAAATTGAAGAGAACGTC |
| PVY5 RP | GGTACCGTGAATGTCCTTGTCTTATTTGC |
| PVY6 FP | GTCGACGAACTTCGGTGCAAAGAGAAG |
| PVY6 RP | GGATCCGTCTCCTGATTGAAGTTTACAGTCAC |
| NIaProFP | GGGGACAAGTTTGTACAAAAAAGCAGGCTCCATGACCATTCATGCTTACTTCAGG |
| NIaProRP | GGGGACCACTTTGTACAAGAAAGCTGGGTTTTGCTCTACAACAACATCATGATC |
| IAASFP | GGGGACAAGTTTGTACAAAAAAGCAGGCTCCATGAAAACGGTGGCAGAGAAAAC |
| IAASRP | GGGGACCACTTTGTACAAGAAAGCTGGGTTACAGAATGCAGTACTAAAATAG |
| SiIAASFP | GGGGACAAGTTTGTACAAAAAAGCAGGCTCATTGAAAGGTAAAAACTTTG |
| SiIAASRP | GGGGACCACTTTGTACAAGAAAGCTGGGTATGAAAACGGTGGCAGAGA |
| SiPDSFP | GGGGACAAGTTTGTACAAAAAAGCAGGCTCCAAGCATTGCTGGCAAGAG |
| SiPDSRP | GGGGACCACTTTGTACAAGAAAGCTGGGTGACAATACAGTTAACTATTTG |
| PExIAASFP | ATCGATATGAAAACGGTGGCAGAG |
| PEXIAASRP | GTCGACTCAACAGAATGCAGTACTA |
| qPVYFP | ACGTCCAAAATGAGAATGCC |
| qPVYRP | TTCAATGCACCAAACCATAA |
| qARF1FP | GGAACTCTGGCATGCCTGT |
| qARF1FP | GCTGGCAAATTGAAAGAAGGA |
| qARF3FP | GGTGTGTATGGAGCTGTGG |
| qARF3RP | AACGTGAGGAGGGAGGTTAT |
| qSAUR3FP | ATGGCTATTCGTATGCCTCG |
| qSAUR3RP | GCAAGTCTTGAAATAAAGGTTG |


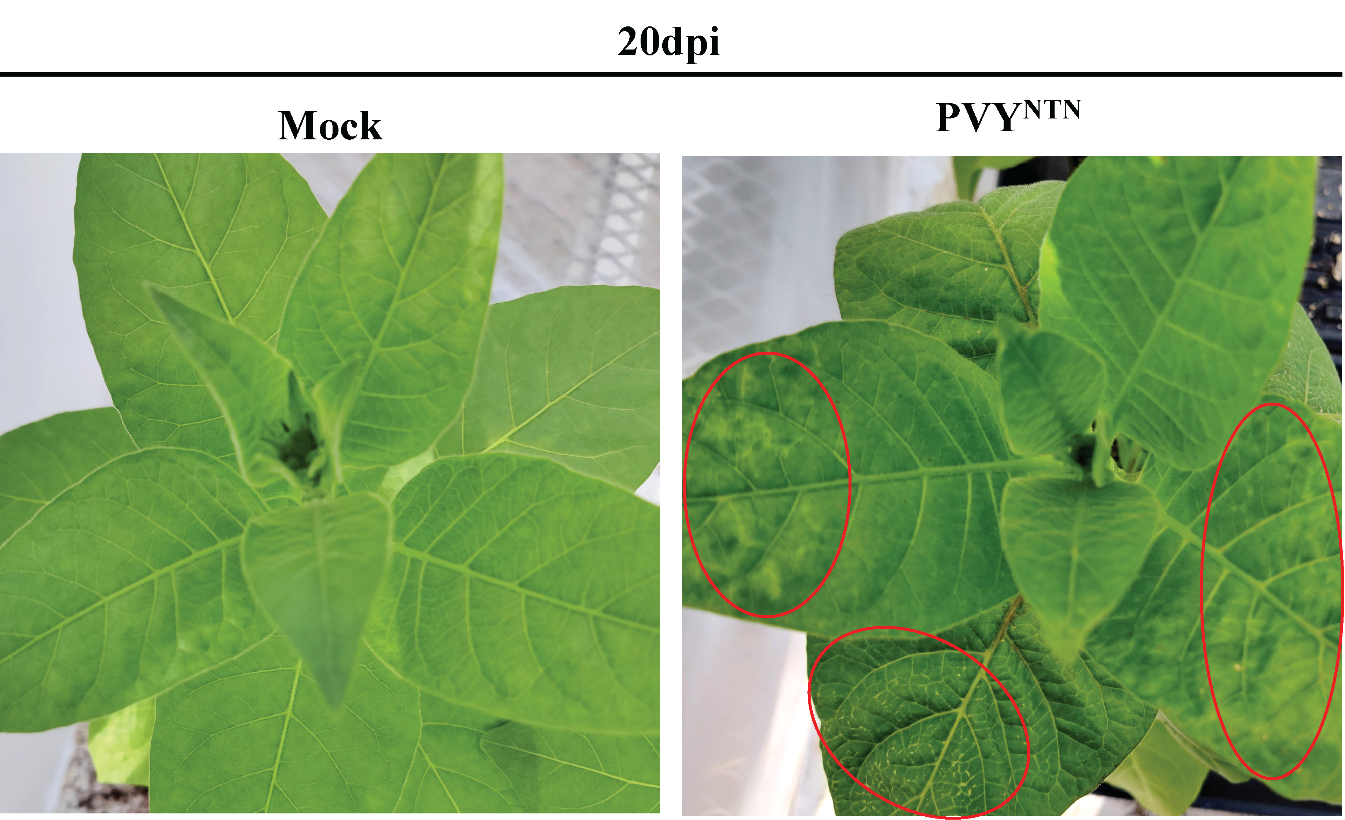
**Supplementary Figure 1: Potato virus Y tuber necrotic strain (PVY^NTN^)-mediated symptom induction on *Nicotiana tabacum.***

Photograph of mock- or PVY^NTN^-inoculated *Nicotiana tabacum* plants at 20 days post-inoculation (dpi). Chlorotic mosaic symptoms developed on the third, fourth, and fifth systemically infected leaves of the PVY^NTN^ inoculated plants are encircled.


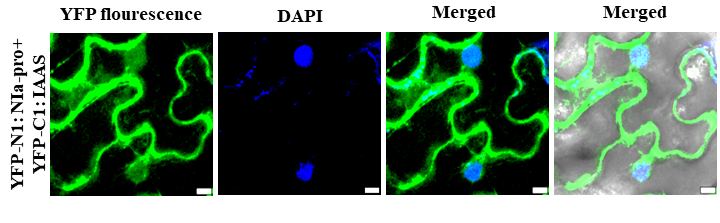
**Supplementary Figure 2: Potato virus Y encoded- NIa-pro protein interacts with *Nicotiana benthamiana* *Indole-3-acetic acid-amido synthetase* (*IAAS*).**

The reconstituted yellow fluorescent protein (YFP) fluorescence was observed in the leaves co-infiltrated with YFP-N1:NIa-pro and YFP-C1:IAAS. Image in panels 1, 2, 3, and 4 are showing YFP fluorescence image, DAPI fluorescence image, merged YFP and DAPI fluorescence image, and merged brightfield with YFP and DAPI fluorescence image. Scale bar represents 50 µM.

**
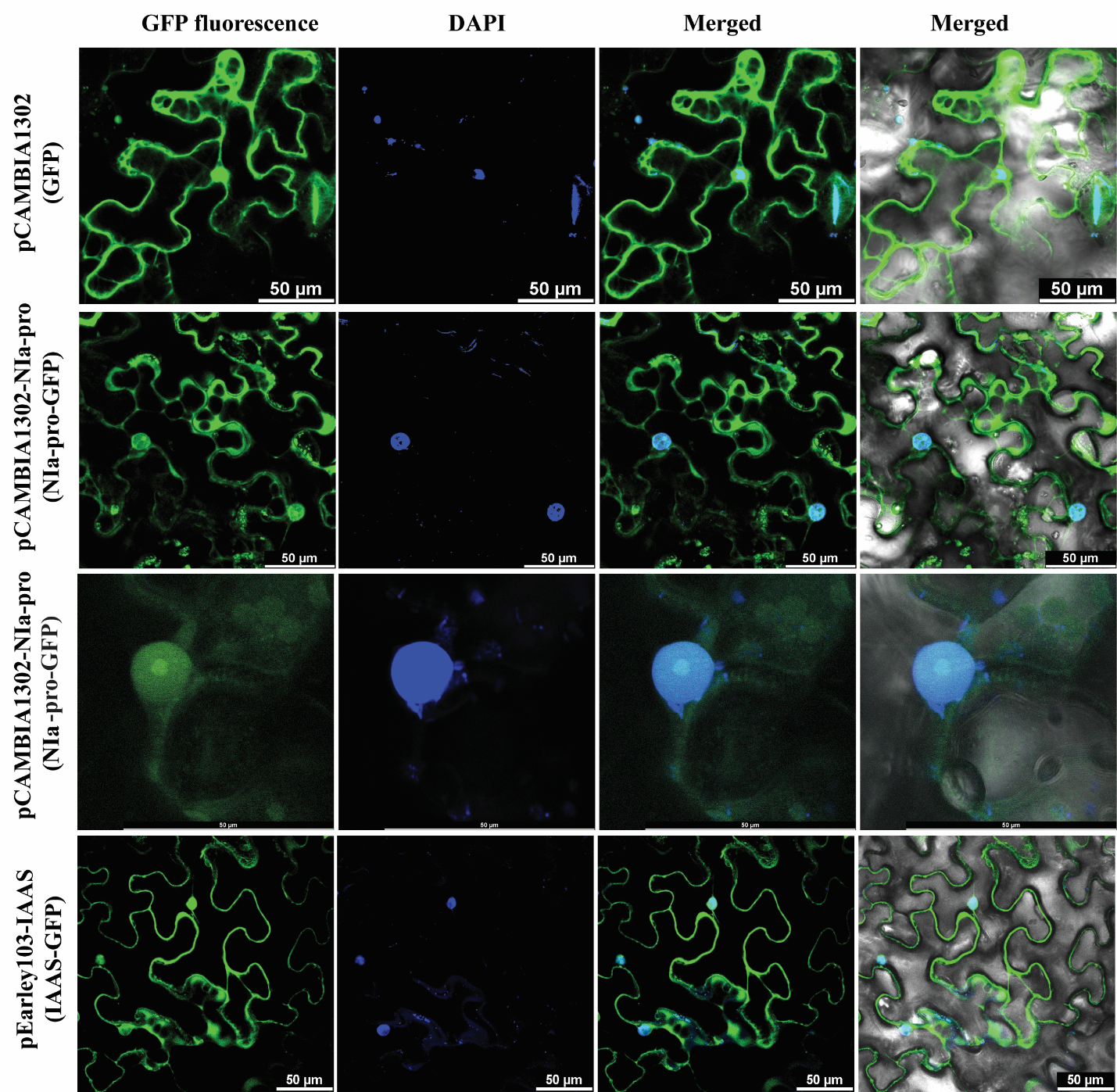
Supplementary Figure 3: Subcellular localization of NIa-pro of potato virus Y and *Indole-3-acetic acid-amido synthetase* proteins.**

Agrobacterium cells harboring either pCAMBIA1302 vector, pCAMBIA1302-NIa-pro or pEarley103-IAAS expression constructs were infiltrated into the leaves of 3- to 4-week-old *Nicotiana benthamiana* plants. The expression of free green fluorescent protein (GFP), NIa-pro-GFP, and IAAS-GFP in the abaxial epidermal cells of infiltrated *N. benthamiana* leaves were visualized under confocal microscope at 48 hours post-infiltration. The nuclei of the epidermal cells were stained with 4, 6-diamidino-2-phenylindole (DAPI). Scale bar represents 50 µM.


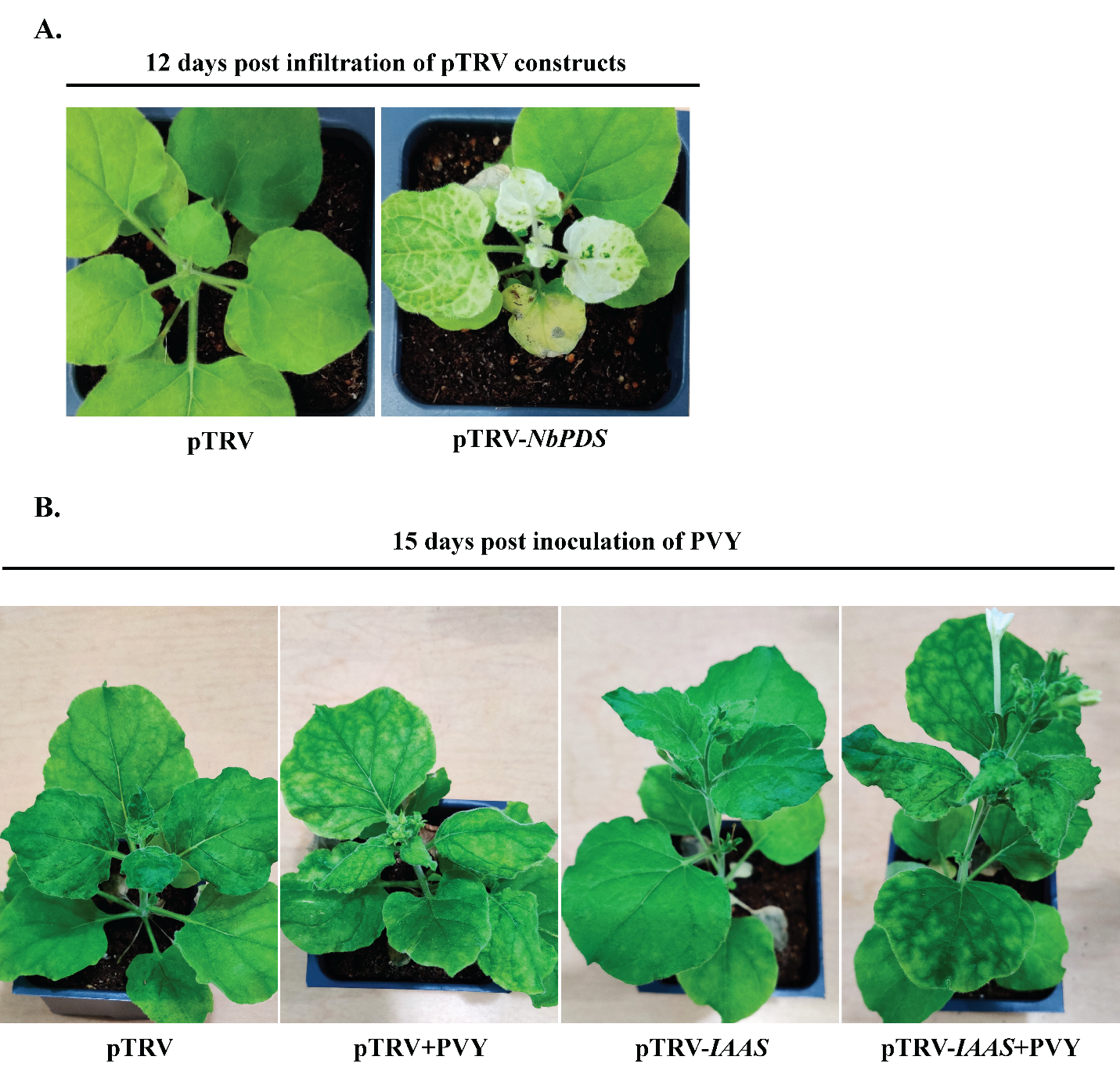


**Supplementary Figure 4: Silencing of *Indole-3-acetic acid-amido synthetase* (*IAAS*) reduces the potato virus Y (PVY)-mediated symptom induction in *Nicotiana benthamiana*.**

(A) Photograph of *Nicotiana benthamiana Phytoene desaturase* (*NbPDS*)*-*silenced and the tobacco rattle virus (pTRV)-infiltrated control *N. benthamiana* plants at 12 days post-infiltration (dpi). (B) Photograph showing mock-inoculated pTRV-infiltrated control plant (pTRV), PVY-inoculated pTRV-infiltrated control plant (pTRV+PVY), mock-inoculated *IAAS*-silenced plant (pTRV-*IAAS*), and PVY-inoculated *IAAS*-silenced plant (pTRV-*IAAS+*PVY) at 15 days post-inoculation.


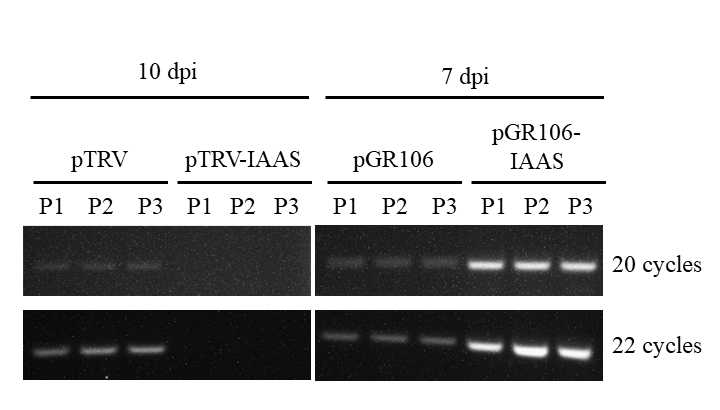
**Supplementary Figure 5: Comparative level of *Indole-3-acetic acid-amido synthetase* (*IAAS*) transcripts in IAAS silenced (pTRV-IAAS) or IAAS overexpressing (pGR106-IAAS) tissues from infiltrated *Nicotiana benthamiana* plants.**

**Left panel is showing s**emiquantitative reverse transcriptase PCR gels showing the comparative level of IAAS transcripts in IAAS-silenced (pTRV-IAAS) or control (pTRV) tissues from infiltrated *N. benthamiana* plants. **Right panel is showing s**emiquantitative reverse transcriptase PCR gels showing the comparative level of IAAS transcripts in IAAS-overexpressing (pGR106-IAAS) or control (pGR106) tissue from infiltrated *N. benthamiana* plants.


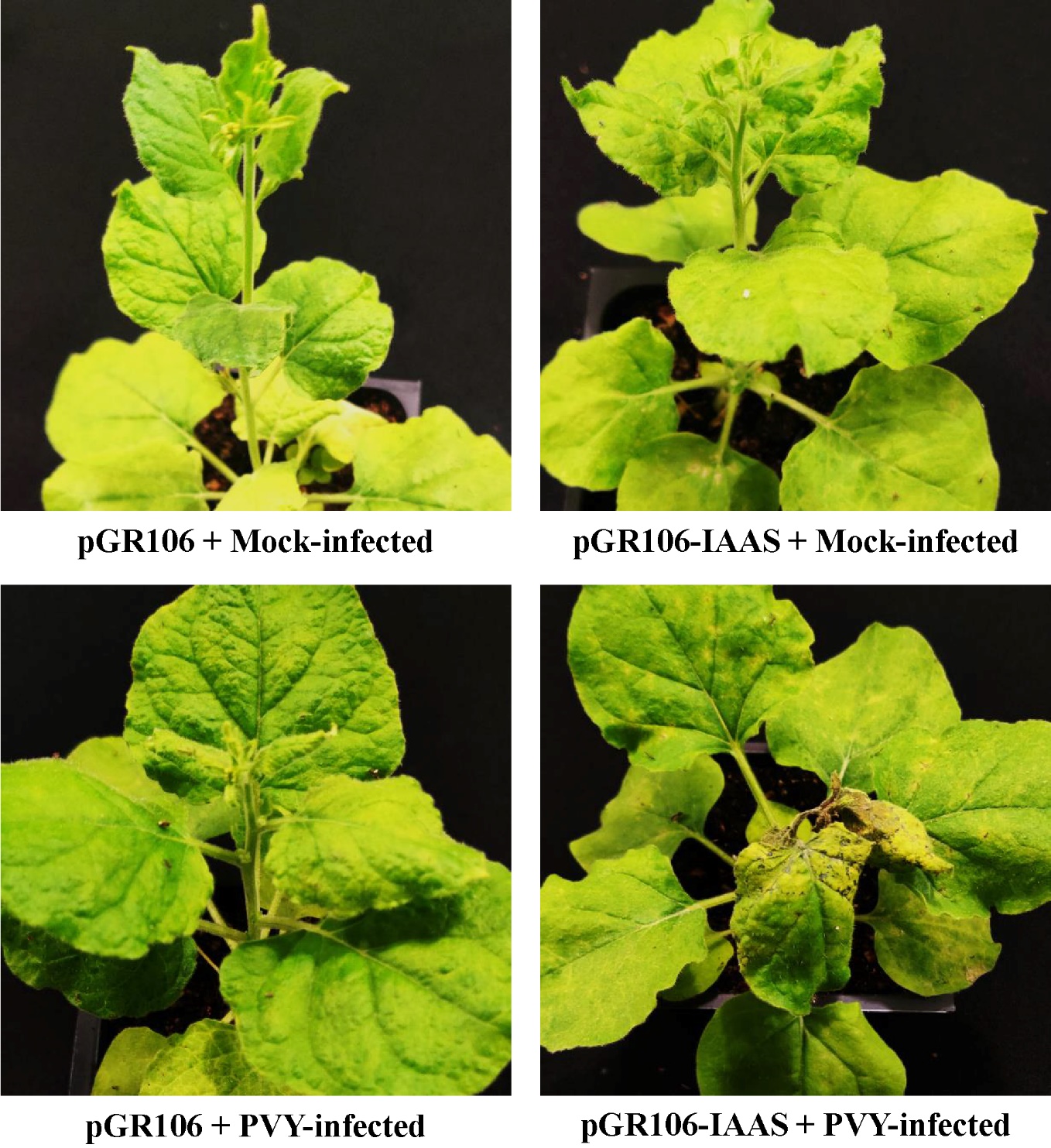
**Supplementary Figure 6: *Indole-3-acetic acid-amido synthetase* (*IAAS*) overexpression aggravate potato virus Y-mediated symptoms.** Photograph showing mock-inoculated pGR106-infiltrated control plant (pGR106 + Mock-infected), PVY-inoculated pGR106-infiltrated control plant (pGR106+PVY-infected), mock-inoculated IAAS overexpressing plant (pGR106-IAAS + Mock-infected), and PVY-inoculated IAAS overexpressing plant (pGR106-IAAS + PVY-infected) at 15 days post-inoculation (dpi).





**Supplementary Figure 7: Relative expression of auxin-responsive genes in the *Nicotiana benthamiana* plants.** Relative expression of selected auxin-responsive genes, Auxin response factor 1 (ARF1), Auxin response factor 3 (ARF3), and small auxin upregulated RNA 3 (SAUR3) in the PVY infected *N. benthamiana* plants at 24 and 48 hours post infiltration (hpi) tested by qRT-PCR. “ns” indicates not significant, p-value > 0.01 as determined by one-way ANOVA.
